# Pharmacokinetics, Pharmacodynamics, Safety, Tolerability, and Immunogenicity of the QX002N anti-IL-17 Monoclonal Antibody: A Phase I, Randomized, Double-Blind, Single Ascending Dose Study in Healthy Chinese Volunteers

**DOI:** 10.3389/fphar.2021.794054

**Published:** 2022-03-04

**Authors:** Min Wu, Hong Zhang, Qianqian Li, Hong Chen, Min Fang, Lizhi Yang, Yanhua Ding

**Affiliations:** ^1^ Department of Phase I Clinical Trial Unit, The First Hospital of Jilin University, Changchun, China; ^2^ Qyuns Therapeutics Co., Ltd., Beijing, China; ^3^ Nanguan District Maternal and Child Health and Family Planning Service Center of Changchun, Changchun, China

**Keywords:** QX002N, IL-17, tolerability, pharmacokinetics, immunogenicity

## Abstract

**Background:** The innovative injection of interleukin 17 A (IL-17A) monoclonal antibody QX002N is being developed to treat active ankylosing spondylitis and plaque psoriasis in adults.

**Objective:** This study investigated the pharmacokinetics (PKs), pharmacodynamics (PDs) safety, tolerability, and immunogenicity of single ascending subcutaneous injections of QX002N in healthy Chinese volunteers.

**Methods:** A total of 65 healthy subjects were enrolled in a randomized, double-blind, placebo-controlled, single ascending dose phase I study (10–320 mg). Ten subjects were allocated to each cohort (containing 8 subjects treated with QX002N and 2 with placebo), except cohort 1 (only 4 subjects treated with QX002N and 1 with placebo). The studies on PKs, PDs, tolerability, and immunogenicity of QX002N were performed.

**Results:** Our study showed that QX002N injection was well tolerated, without deaths, serious adverse events, or discontinuations due to treatment-emergent adverse events (TEAEs). Neither more frequency nor high severity of the drug-related adverse reaction was observed with increasing QX002N dose. The TEAEs in all subjects were considered Grades 1–2 (CTCAE 5.0) except for one case of Grade 3 (hypertriglyceridemia). T_max_ of QX002N was obtained from 168 to 240 h across the dose range after administration. The C_max_ and area under the curve of QX002N increased in proportion to dose, and showed linear PKs. Anti-drug antibody positivity was detected in one (1.9%) subject after drug administration.

**Conclusion:** QX002N was well tolerated in our study. Based on the PKs and safety results of QX002N, 80 mg is recommended as the effective dose for a future phase Ib study.

**Clinical Trial Registration**: https://www.chinadrugtrials.org.cn/, identifier ChiCTR1900023040.

## Highlights

1. QX002N injection is a first-in-class, anti-IL-17A monoclonal antibody, which was evaluated in a first-in-human, Phase I, SAD in healthy Chinese subjects regarding its PKs, PDs, safety, tolerability, and immunogenicity.
2. The C_max_ and AUC of QX002N increased in a dose-proportional manner, and showed linear PK characteristics.
3. The PKs and safety profiles of QX002N indicated that 80 mg could be administered as an effective dose for a future phase Ib study. The results of this Phase I trial in healthy subjects warrant the study of QX002N as a therapeutic for patients with psoriasis and ankylosing spondylitis in subsequent phase Ib and phase II studies.

## Introduction

Interleukin 17A (IL-17A) is a naturally occurring cytokine that links T-cell activation to neutrophil mobilization and activation, and is one of the principal pro-inflammatory cytokines in some autoimmune diseases. Therefore, the neutralization of IL-17A is considered a therapeutic approach for immune-mediated diseases, such as inflammation and tissue destruction, as well as symptom relief (Blauvelt, 2008; Krueger et al., 2012; Lynde et al., 2014).

The role of T helper 17 (Th17) cells and other immune cells, as proximal regulators of psoriatic skin inflammation, has recently been reported. IL-17A, a principal effector cytokine of Th17 cells, stimulates keratinocytes to produce chemokines, cytokines, and other proinflammatory mediators, thereby enabling IL-17A to bridge the innate and adaptive immune systems to sustain chronic inflammation. Therefore, inhibition of the IL-17 pathway may provide a new therapeutic approach for patients with moderate to severe psoriasis and ankylosing spondylitis (AS) (Lynde et al., 2014; Chyuan and Chen, 2018).

Currently, the therapies for AS and psoriasis are still limited, including nonsteroidal anti-inflammatory drugs (NSAIDs), hormone therapy, and immunosuppressive drugs. However, very few medicines (such as anti-tumor necrosis factor (TNF) agents, which are approved for clinical use) are available for patients who do not sufficiently respond to NSAID therapy or for patients in whom NSAIDs cause intolerable side effects (Lata et al., 2019). Although the curative effects of anti-TNF agents are generally good, some patients with serious infections, tuberculosis, opportunistic infections, risk of malignancies, and anti-drug antibodies do not respond well (Minozzi et al., 2016).

Specific targeting of the IL-23/Th17/IL-17 axis is a proven therapeutic approach with high efficacy in immune-mediated diseases. For example, Il-17A inhibitors can significantly improve the symptoms, signs, physical function, and quality of life of patients with AS and psoriasis. It is predicted that the market prospect of Il-17A inhibitors is broad.

Among the development of IL-17A inhibitors, QX002N, an innovative first-in-class biological product, is a high affinity recombinant humanized IgG1 monoclonal antibody against human IL-17A. QX002N was produced using Chinese hamster ovary cells and supplied by Qyuns Therapeutics Co., Ltd. (Taizhou, Jiangsu, China). QX002N injection is mainly designed for active AS and moderate to severe plaque psoriasis in adults.

Although Secukinumab and Ixekizumab have been approved for patients with AS and psoriasis, however, the uniqueness and novelty of QX002N are found in comparison to Secukinumab and Ixekizumab. For the pharmacodynamics (PDs), the binding activity of QX002N with human IL-17AA (ranged from 9.51 to 13.5 ng/ml), was comparable to that of Ixekizumab (21.1 ng/ml) and slightly better than that of Secukinumab (83.1 ng/ml); Moreover, the affinity between QX002N and human IL-17AA was 3.38–5.16 pM, which was comparable to Ixekizumab (16.3 pM), but superior to Secukinumab (535 pM).

A PD study *in vitro* and *in vivo* demonstrated that QX002N can specifically bind to human IL-17A, thereby preventing IL-17 AA or IL-17 AF from binding to its receptor (IL-17RA). As we know, the human IL-17A can recognize mouse IL-17A receptors, thereby inducing the release of keratinocyte-derived cytokine (KC) in mice, leading to an increase in serum KC concentration. For the pharmacokinetic (PK) study in mice, different doses of QX002N were intravenously administrated, followed by subcutaneous injection of the same dose of human IL-17A. The evaluation of the neutralizing activity of QX002N against human IL-17A was performed by measuring the content of KC in mouse serum. Our study showed that QX002N can inhibit KC releases induced by human IL-17A (in a dose-dependent manner) in mice.

PK studies of QX002N in animal models have been performed. For example, after different single subcutaneous (SC) doses of QX002N (1.5, 5, 15 mg/kg) in rhesus monkeys, the maximum serum concentration (C_max_) and area under the serum concentration time curve (AUC_0-t_, AUC_0-inf_) of QX002N were increased in proportion to dose. No anti-drug antibodies (ADAs) were observed in the three cohorts (unpublished data). Another study showed that after multiple intravenous injections of QX002N (15, 50, 150 mg/kg) for 4 weeks (once a week) in rhesus monkeys, no animal death was observed. Moreover, in this animal study, all examinations including clinical observations, weight, food intake, vital signs, electrocardiogram (lead II) test, ophthalmology examination, and clinical laboratory tests did not show drug-related abnormalities. No central nervous system toxicity was observed in Sprague–Dawley rats after single intravenous injections of QX002N (15, 50, 150 mg/kg). In New Zealand white rabbits, there was no stimulation reaction upon SC injection, intravenous injection, or intramuscular injection of QX002N or after multiple intravenous injections of QX002N (100 mg/ml) in the ear vein (three times, once every 2 days).

Taken together, the animal studies showed good safety, efficacy, and preclinical PK characteristics of QX002N injection, indicating that QX002N injection is a viable candidate for clinical application as an anti-IL-17A monoclonal antibody to treat psoriasis and AS. The aim of this study was to evaluate the PKs, PDs, safety, tolerability, and immunogenicity after single ascending doses (SADs) of QX002N in healthy Chinese individuals.

## Materials and Methods

### Subjects

All healthy volunteers, aged 18–50 years old, body weight ≥45 kg (female) and ≥50 kg (male), and body mass index values of 18–28 kg/m^2^, were eligible to participate in the study. The main exclusion criteria were as follows: 1) clear history of disease in the central nervous system, cardiovascular system, kidney, or liver or other prominent diseases; 2) abnormal electrocardiogram results, vital sign measurements, clinical laboratory tests, or imagological examination (chest X-ray and ultrasonography); 3) infection with hepatitis B virus, hepatitis C virus, or human immunodeficiency virus; 4) systemic or local infection within 8 weeks of the study screening; 5) severe drug or food allergies, or hypersensitivity to any biologic therapy or vaccine; and 6) consumption of alcohol or alcohol-containing drinks within 24 h before receiving the testing medicine.

### Drugs

QX002N is an IL-17A monoclonal antibody for clinical use (specification: 100 mg/1 ml), which was developed and supplied by Qyuns Therapeutics Co., Ltd. In our study, the recruited subjects received QX002N injection with the same lot number.

### Study Design and Administration

Our study was a phase I, randomized, double-blind, placebo-controlled, SAD clinical trial, which was conducted in the Phase I Clinical Trial Unit of the First Hospital of Jilin University (Changchun City, Jilin Province, China). The clinical study protocol was approved by the Ethics Committee at the Jilin University First Affiliated Hospital-Clinical Research Institute (Approval No. 19Y032-002). Written informed consent was obtained from each participant before the study commenced. There were seven SAD cohorts in this study treated with the following doses of QX002N: 10, 20, 40, 80, 160, 240, and 320 mg by SC injection. The participants (65 subjects) were allocated into each treatment group/cohort (10 subjects/per group including 8 with QX002N and 2 with placebo, at a 4:1 ratio), except for the first cohort (only 4 subjects treated with QX002N and 1 with placebo). The participants in the different cohorts received either the corresponding dosage of QX002N or placebo via SC injection (50 mg/0.5 ml) on the first day.

### Tolerability Measurements

During the period of drug administration until the end of the study (Day 85 or 127), all adverse events (AEs) were recorded. In this study, the definition of treatment-emergent AEs (TEAEs) was based on the National Cancer Institute Common Terminology Criteria for the Classification of Adverse Events (NCI-CTCAE, 5.0). Changes in vital signs (body temperature, sitting blood pressure and heart rate), physical examinations, electrocardiograms, and clinical laboratory tests were also monitored. Safety outcome measures included the nature, frequency, severity, timing, clinical outcome, and association of the test drug with TEAEs. Moreover, escalation to the next dose was performed only after the safety follow-up was completed.

### PK Assessment

To characterize the PK profile of QX002N injection with different SC dosing, serum samples were collected from two cohorts (10 and 20 mg) at different time points after drug administration, including on Day 1 (0, 4, 12), Day 2 (24 h), Day 3 (48 h), Day 4 (72 h), Day 5 (96 h), Day 6 (120 h), Day 8 (168 h), Day 11 (240 h), Day 15 (336 h), Day 22 (504 h), Day 29 (672 h), Day 43 (1008 h), Day 57 (1344 h), Day 71 (1680 h), and Day 85 (2016 h). For the 40 mg cohorts, additional collection time points on Day 106 (2520 h) and Day 127 (3024 h) were added, because the serum concentrations of QX002N in the 10 and 20 mg cohorts were not very low at the last collection timepoint (Day 85). Moreover, for the cohorts treated with ≥80 mg, the time points for blood collection were modified by omitting 12 h and Day 4 (72 h) and adding Day 106 (2520 h) and Day 127 (3024 h). The collected blood samples were placed at room temperature for 30 min, followed by centrifuging at 1800 *g* at 4°C for 15 min. The supernatant was collected and stored at −80°C until PK analysis. Serum concentrations of QX002N at different time points were measured to calculate the PK parameters such as C_max_, time to maximum serum concentration (T_max_), AUC_0-t_ and AUC_0-inf_, λ_z_, mean retention time, clearance (CL/F), volume of distribution (V_z_/F), and terminal elimination half-life (t_1/2_). Non-compartmental methodology (by WinNonlin® Enterprise software, version 8.3.1) was used to calculate the PK parameters. The serum concentration of QX002N was measured using the enzyme-linked immunosorbent assay (ELISA) at United-Power Pharma Tech Co., Ltd. (Beijing, China). The calibration range of QX002N was 25–2000 ng/ml. The lower limits of quantification of the assay for QX002N were 25 ng/ml. The accuracy of the assay for QX002N was 0–2.2%, and the precision was within 11.3% coefficient of variation. SAS software (version 9.4) was used for Linear dose assessment of C_max_ and AUC of QX002N after a single dose by Linear Mixed Effect Model.

### Immunogenicity Evaluation

The bridging electrochemiluminescence assay method was used to detect ADAs in the serum samples that were collected on Day 1 (before drug administration), and on Days 15, 29, 57, and 85 for both the 10 and 20 mg cohorts. For the 40 mg dose cohort on Day 127 (3024 h), an additional time point was added. For the ≥80 mg dose cohort, the time points for blood collection were modified by omitting Day 15 (336 h) and adding Day 127 (3024 h). ADA assays were performed using the Bridging-ECLA at United-Power Pharma Tech Co., Ltd. (Beijing, China). The ADA test is a hierarchical test, which includes a screening test, confirmation test, and titer test. The sensitivity of the screening test and confirmation test was 1.9 and 3.0 ng/ml, respectively. The drug tolerance for the ADA assay was also assessed in our study. The data showed that the ADA levels of 20, 100, and 250 ng/ml were reproducibly detectable in the presence of 50, 200 and 200 μg/ml QX002N, respectively, which were much higher than the levels of drug in the serum sample for the immunogenicity study.

### PD Evaluation

The serum levels of IL-6 and IL-8 of all cohorts were measured by ELISA using the collected blood samples at different time points, including Day 1 (before administration), and Days 6, 15, 22, and 43. Skanit Software 4.1 was used for the analysis of the ELISA results, including absorbance reading, basic calculation, and standard curve fitting. In addition, Excel 2010 was also used for the data analyses. The concentrations of IL-6 and IL-8 were measured using the ELISA at the Institute of Translational Medicine of First Hospital of Jilin University (Changchun, China). The levels of IL-6 and IL-8 at 0 h (before administration) were used as a baseline to compare the differences in concentrations of the two cytokines between different time points at baseline and after administration.

## Results

### Demographics

As shown in Table 1 65 subjects (52 in the QX002N group, 13 in the placebo group) were enrolled in this study. Table 1 summarizes the subject demographics of the different dose cohorts.

**TABLE 1:** Summary of the demographic characteristics.

Characteristic	QX002N							Placebo (N = 13)	Total (N = 65)
	10 mg (N = 4)	20 mg (N = 8)	40 mg (N = 8)	80 mg (N = 8)	160 mg (N = 8)	240 mg (N = 8)	320 mg (N = 8)		
Sex, *n* (%)									
Male	2 (50)	4 (50)	5 (62.5)	4 (50)	5 (62.5)	4 (50.0)	3 (37.5)	6 (46.2)	34 (51.5)
Female	2 (50)	4 (50)	3 (37.5)	4 (50)	3 (37.5)	4 (50.0)	5 (62.5)	7 (53.8)	32 (48.5)
Age, years	38.3 (1.7)	33.1 (7.9)	30.3 (6.0)	36.0 (8.1)	39.4 (9.8)	38.4 (6.3)	39.8 (7.6)	35.9 (6.6)	36.1 (7.5)
Weight (kg)	67.4 (13.6)	63.5 (10.2)	69.2 (8.1)	59.6 (3.7)	64.0 (7.3)	66.0 (6.9)	60.3 (6.9)	58.9 (9.5)	63.1 (8.6)
Height (cm)	162.5 (11.9)	162.8 (9.5)	167.5 (6.4)	162.4 (8.6)	163.8 (8.2)	165.6 (6.1)	160.7 (7.4)	160.3 (7.3)	163.1 (7.8)
BMI (kg/m^2^)	25.3 (2.4)	23.9 (2.4)	24.6 (2.0)	22.9 (3.1)	23.9 (2.4)	24.0 (2.2)	23.5 (2.9)	22.8 (2.1)	23.7 (2.4)

Abbreviations: BMI, body mass index; *Data are mean (SD) unless otherwise noted.

### Tolerability Measurement

In this study, a total of 91 clinical TEAEs were reported in 37 (57%) randomized subjects, of whom 78 clinical TEAEs were reported in 31 (59.6%, 31/52) of the randomized subjects in the QX002N group. Furthermore, 65 cases of TEAEs in 30 subjects were reported as adverse drug reactions (ADRs). All ADRs in the different cohorts are summarized in Table 2; the data showed that there were no clear dose-related trends. All ADRs occurring in this study were accidental, because neither the ADR frequency nor severity increased with increasing QX002N dose. The ADRs in all subjects were considered Grade 1–2 (CTCAE 5.0), with the exception of one subject (No.1003) in the 10 mg cohort, who developed “hypertriglyceridemia” belonging to CTCAE Grade 3. This subject recovered 5 days after reporting TEAEs, without any medical intervention. The most frequently reported TEAEs (≥5%) related to the administration of QX002N injection were increased bilirubin (7.7%), increased aspartate aminotransferase (7.7%), decreased leukocytes (6.2%), decreased neutrophils (6.2%), urinary tract infection (10.8%), and urinary erythrocytes (7.7%). Most of the TEAEs were caused by infection, perhaps related to the drug’s mechanism of action. There were no TEAEs of erythema or swelling at the injection site. There were also no deaths, serious AEs, or drug discontinuation due to TEAEs.

**TABLE 2:** Type and incidence of ADRs reported.

	10 mg (N = 4)	20 mg (N = 8)	40 mg (N = 8)	80 mg (N = 8)	160 mg (N = 8)	240 mg (N = 8)	320 mg (N = 8)	Placebo (N = 13)	Total (N = 65)
Subjects with at least one ADR	3 (75.0%)	7 (87.5%)	5 (62.5%)	5 (62.5%)	2 (25.0%)	5 (62.5%)	3 (37.5%)	4 (30.8%)	34 (52.3%)
Bilirubin increasing	0	1 (12.5%)	0	1 (12.5%)	1 (12.5%)	1 (12.5%)	0	1 (7.7%)	5 (7.7%)
Aspartate aminotransferase increasing	0	1 (12.5%)	2 (25.0%)	0	0	0	0	2 (15.4%)	5 (7.7%)
Leukocyte decreasing	0	1 (12.5%)	0	1 (12.5%)	0	2 (25.0%)	0	0	4 (6.2%)
Neutrophils decreasing	0	1 (12.5%)	0	1 (12.5%)	0	2 (25.0%)	0	0	4 (6.2%)
Alanine aminotransferase increasing	0	0	1 (12.5%)	0	0	0	0	1 (7.7%)	2 (3.1%)
Blood creatinine increasing	0	0	0	0	0	0	1 (12.5%)	0	1 (1.5%)
urinary cast increasing	0	0	0	1 (12.5%)	0	0	0	0	1 (1.5%)
Leukocyte increasing	0	0	1 (12.5%)	0	0	0	0	0	1 (1.5%)
Platelet counts decreasing	0	0	1 (12.5%)	0	0	0	0	0	1 (1.5%)
Neutrophil increasing	0	0	1 (12.5%)	0	0	0	0	0	1 (1.5%)
Total bile acid increasing	0	0	0	0	0	0	0	1 (7.7%)	1 (1.5%)
Hyperuricemia	0	1 (12.5%)	0	0	0	1 (12.5%)	1 (12.5%)	0	3 (4.6%)
Hyperglyceridemia	1 (25.0%)	0	1 (12.5%)	0	0	1 (12.5%)	0	0	3 (4.6%)
Hyperkalemia	0	1 (12.5%)	0	0	0	1 (12.5%)	0	0	2 (3.1%)
Hyperglycemia	0	0	0	1 (12.5%)	1 (12.5%)	0	0	0	2 (3.1%)
Hypercholesteremia	0	1 (12.5%)	0	0	0	0	0	0	1 (1.5%)
Urinary tract infection	1 (25.0%)	0	0	2 (25.0%)	0	1 (12.5%)	0	3 (23.1%)	7 (10.8%)
Upper Respiratory Infection	1 (25.0%)	0	1 (12.5%)	0	0	0	0	1 (7.7%)	3 (4.6%)
Urinary erythrocyte positive	0	1 (12.5%)	1 (12.5%)	2 (25.0%)	0	0	1 (12.5%)	0	5 (7.7%)
Proteinuria	0	0	1 (12.5%)	0	0	1 (12.5%)	0	0	2 (3.1%)
Diarrhea	0	2 (25.0%)	0	0	0	0	0	0	2 (3.1%)
Nausea	0	1 (12.5%)	0	0	0	0	0	0	1 (1.5%)

Abbreviations: ADR, adverse drug reaction; N, number ADRs; n%, incidence of subjects reporting ADRs.

### PK Analyses

**FIGURE 1:**
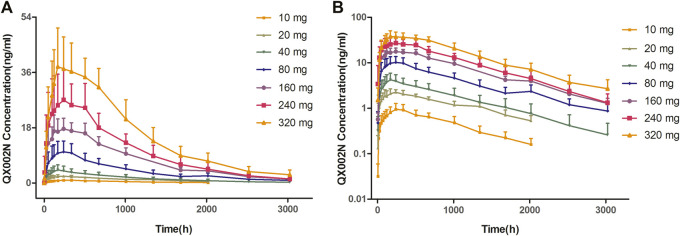
Mean concentration–time curve with standard deviation after subcutaneous injection of a single ascending dose of QX002N in healthy Chinese volunteers.

After a single SC dose of QX002N at different concentrations, ranging from 10 to 320 mg (Figure 1), the C_max_ and AUC of QX002N were increased in a dose-proportional manner, and showed a linear PK (Figures 1A,B). Moreover, the increasing proportion of AUC was similar to the increasing proportion of the dose (β1 was 1.04–1.08). The T_max_ of QX002N was calculated using the data from the serum samples of different dose cohorts, which were collected from 168 to 240 h after drug administration (Table 3). The mean terminal phase elimination half-life (t_1/2_) of QX002N was estimated for the serum samples of different dose cohorts collected between 586 and 843 h after drug administration. PK analyses also showed that QX002N was absorbed and eliminated slowly after its administration. Finally, other PK parameters of QX002N including T_max_, t_1/2_, V_z_/F, and CL/F were not statistically significant across the dose range.

**TABLE 3:** Mean (SD) pharmacokinetic parameters for QX002N in healthy subjects after single ascending dosing of QX002N injection.

Parameter	10 mg (N = 4)	20 mg (N = 8)	40 mg (N = 8)	80 mg (N = 8)	160 mg (N = 8)	240 mg (N = 8)	320 mg (N = 8)
T_max_ (h)	288 (240, 336)	240 (96, 504)	168 (168, 504)	240 (120, 336)	288 (48, 504)	240 (96, 504)	204 (168, 504)
C_max_ (μg/ml)	0.98 (0.29)	2.42 (0.33)	4.39 (1.57)	10.53 (3.52)	19.69 (4.35)	29.76 (9.77)	41.75 (7.70)
AUC_0-t_ (h*μg/mL)	955 (301)	2574 (350)	4521 (1830)	11345 (3499)	22501 (4053)	30407 (8801)	46674 (9837)
AUC_0-inf_ (h*μg/mL)	1116 (360)	3122 (540)	4787 (2027)	12500 (4049)	23723 (4447)	31593 (9018)	49485 (11062)
λ_z_ (h^−1^)	0.001 (0)	0.001 (0.0002)	0.0012 (0.0003)	0.0009 (0.0003)	0.0011 (0.0001)	0.0012 (0.0002)	0.0011 (0.0003)
t_1/2_ (d)	29.3 (0.3)	29.0 (6.3)	24.4 (6.5)	35.0 (9.1)	27.7 (4.5)	25.2 (5.1)	27.0 (7.7)
Vz/F (L)	10.0 (4.0)	6.5 (1.5)	8.4 (4.5)	8.3 (2.6)	6.5 (1.1)	7.3 (3.3)	6.2 (1.5)
CL/F (L/h)	0.0099 (0.004)	0.0066 (0.0011)	0.0109 (0.0078)	0.0073 (0.0036)	0.007 (0.0013)	0.0084 (0.0035)	0.0068 (0.0018)
MRT (h)	1077 (37)	1189 (208)	1000 (214)	1236 (255)	1088 (105)	995 (234)	1073 (223)

Data are shown as geometric mean (SD), except T_max_ which are as median (Min–Max).

### Evaluation of Drug Immunogenicity

Overall, ADA positivity was detected only in one subject (1.9%) in the 20 mg cohort on Day 85 after drug administration. Another subject in the placebo group was found in the ADA-positive group on Day 29, which was still positive until the last ADA test (Day 127). The other subjects in this study were ADA-negative, suggesting that after drug administration, the possibility of ADAs is very low.

### Analysis of PDs

PD analyses showed that after a single dose of QX002N, the change in serum concentration of IL-6 was not clinically significant compared to baseline. Also, there was no significant correlation between changes in IL-6 concentration and drug dose. Although IL-6 and IL-8 levels decreased or increased in individual subjects in all dose cohorts, there was no significant trend. Thus, the variation in serum concentration of IL-6 and IL-8 in healthy individuals may not have clinical value for evaluating QX002N injection.

## Discussions

This study was a phase I, randomized, double-blind, placebo-controlled, SAD clinical trial, which determined the safety, tolerability, and PK profiles of QN002 injection after administration of single SC doses in healthy adult subjects.

QN002N was safe and well-tolerated in our study. The most frequently reported TEAEs related to drug administration were high bilirubin and aspartate aminotransferase levels, low leukocyte and neutrophils counts, urinary tract infection, and urinary erythrocytes. Most of the TEAEs were associated with infections, consistent with the literature (Gomez et al., 2017; Bruin et al., 2020). However, in our study, the incidence of TEAEs in each category was lower than 15%, as compared to that of similar drugs in the literature (Ellis et al., 2019), in which the incidences of nasopharyngitis and headache were more than 50%, suggesting that the safety of QN002N is better than that of other monoclonal antibodies.

The maximal recommended starting dose (MRSD) of QX002N injection was determined by calculating the no-observed-adverse-effect-level (NOAEL) exposure per regulatory guidelines (FDA, 2005) and the rule of the first clinical starting dose of the same drug class. In a previous report, the NOAEL dose for QX002N in a 4-week, repeat-dose cynomolgus monkey toxicity study was 150 mg/kg; the safety factor was 10-fold and the MRSD was 364 mg. For example, the starting dose of intravenous administration of secukinumab was at 0.1 mg/kg for treating patients with AS (European Medicines Agency, 2015). If a patient’s body weight is 60 kg, the converted SC dose is about 7.5 mg. In another example, the starting dose of SC administration of ixekizumab was 5 mg for treating patients with psoriasis vulgaris (Australian Government Department of Health Therapeutic Goods Administration (TGA), 2021). In addition, the preparation specifications and convenience of the operation of the investigational drug were also considered. Therefore, the optimal MRSD of QX002N was 10 mg. The maximum dose of QX002N was calculated based on a 4-week, repeat-dose cynomolgus monkey toxicity NOAEL exposure (C_max_ and AUC). The highest dose of 320 mg SC had a safety factor of at least 20-fold or greater.

After single SC dosing of QX002N at different doses, ranging from 10 to 320 mg, the value of C_max_ and AUC of QX002N was increased in proportion to the dose of QX002N, showing a linear PK. This result is consistent with the PK characteristics of the preclinical animal test.

According to the analysis of the Pop PK study, patients with chronic psoriasis vulgaris received ixekizumab (FDA-approved anti-IL-17 monoclonal antibody), of which the first time SC dose was 150 mg in the Phase I study (Study RHAG). The geometric mean of C_max_ and AUC_(0–14days)_ of ixekizumab were 8.19 ± 0.04 μg/ml and 95.1 ± 39 day*μg/mL, respectively. Another example was a Phase III, multicenter, randomized, open-label, parallel-group, 12-week study for treating the patients with moderate to severe plaque psoriasis (Study RHBL). The primary PK parameters of ixekizumab were C_max_ 12.7 (11.3, 14.2) μg/ml and AUC_0-14d_ 135 (122,150) day*μg/mL, following the starting dose of 160 mg SC dose (Australian Government Department of Health Therapeutic Goods Administration (TGA), 2021). In our study, the C_max_ and AUC_0-t_ of QX002N in the 80 mg dose cohort were 10.53 ± 3.52 μg/ml and 117.63 ± 45.58 day*μg/mL, respectively. As stated above, the exposure levels (C_max_, AUC_(0–14days)_) after a single SC administration of QX002N (80 mg) in healthy subjects were comparable to those of ixekizumab (160 mg) SC administration in the patients with plaque psoriasis. Therefore, the therapeutic dose (80 mg) of QX002N in the phase Ib study was determined, based on the PK results, which is lower than the ixekizumab dose.

The immunogenicity of biological drugs is a major concern for their clinical application, because of the effects on PK and PD of drugs. In this study, the immunogenicity of QX002N injection was examined by the ADA test. Overall, ADA positivity was detected in one (1.9%) subject in the 20 mg cohort on Day 85 after drug administration. Another subject in the placebo group was found to be ADA-positive on Day 29. Except for these two subjects, all other subjects were ADA-negative, suggesting that the possibility of QX002N-induced ADA after administration is very low.

IL-17Rs are targeted by a group of pro-inflammatory IL-17 cytokines (IL17A-F). In human astrocytes, IL-17A and TNF can upregulate the mRNA expression of IL-6, IL-8, and Th17 cytokines (Elain et al., 2014). Therefore, the levels of IL-6 and IL-8 were selected as indicators of PDs. However, there were no significant changes in the levels of IL-6 and IL-8 after drug administration in our study, possibly because all of the participants in our study were healthy subjects. Healthy people have a normal immune function and high compensatory capacity, which might be the reason that there are no changes of PD markers IL-6 and IL-8 in healthy volunteers. Similar results were found in the other study on the same target (Ellis et al., 2019). No meaningful changes were observed in absolute numbers or proportions of immune cell populations or inflammatory cytokine profiles (IL-6, tumor necrosis factor-α, interferon-γ, and IL-2). It is notable that IL-8 was increased at apparently random time points in some subjects. We will continue to evaluate the clinical value of IL-6 and IL-8 in subsequent patient studies.

A limitation of our study was that the sample size in each study was too small to examine the effect of sex on the PK of QX002N. Second, multiple doses were not included because the half-life of the drug was too long. The study period will be prolonged due to multiple doses, so that subject compliance will decrease, and the rate of withdrawal will increase. Therefore, the evaluation of the tolerance and accumulation of QX002N will be performed in the subsequent phase Ib study for patients.

## Conclusion

In this study, we revealed the linear PK profile of QX002N after an SAD of SC injection in healthy Chinese volunteers. QX002N was generally well tolerated in the healthy subjects over the tested drug dose range. ADA positivity was detected in only one (1.9%) patient after drug administration. The PK and safety profiles of QX002N in healthy Chinese subjects suggest that 80 mg can be an effective dose for a future phase Ib study.

## Data Availability

The original contributions presented in the study are included in the article/Supplementary Material, further inquiries can be directed to the corresponding author.

## Abbreviations

AS, ankylosing spondylitis; IL-17A, anti-interleukin 17A; PDs, pharmacodynamics; PK, pharmacokinetic; SAD, single ascending dose; TEAEs, treatment-emergent adverse events.
